# Parasite burden in a short-lived chameleon, *Furcifer labordi*

**DOI:** 10.1016/j.ijppaw.2019.09.010

**Published:** 2019-09-30

**Authors:** Falk Eckhardt, Christina Strube, Karina A. Mathes, Frank Mutschmann, Hauke Thiesler, Cornelia Kraus, Peter M. Kappeler

**Affiliations:** a Dept. Sociobiology/Anthropology, Institute of Zoology and Anthropology, University of Göttingen, Kellnerweg 6, 37077, Göttingen, Germany; b Institute for Parasitology, Centre for Infection Medicine, University of Veterinary Medicine Hannover, Bünteweg 17, 30559, Hannover, Germany; c Clinic for Small Mammals, Reptiles and Birds, University of Veterinary Medicine Hannover, Bünteweg 9, 30559, Hannover, Germany; d Exomed-Labor, Schönhauser Straße 62, 13127, Berlin, Germany; e Institute of Clinical Biochemistry, Hannover Medical School Carl-Neuberg-Strasse 1, 30625, Hannover, Germany; f Behavioral Ecology and Sociobiology Unit, German Primate Center, Kellnerweg 4, 37077, Göttingen, Germany

**Keywords:** Parasites, Chameleons, Immunosenescence, Aging, *Furcifer*

## Abstract

Life history theory predicts that species with shorter lifespan should show higher investments into growth and reproduction at the expense of immune defenses. Labord's chameleon (*Furcifer labordi*) is the tetrapod with the shortest known life span. To investigate to which extent immunosenescence influences the die-off of these chameleons when they are only about 6 months old, we examined the gastrointestinal-, blood- and ectoparasite burden in *F. labordi* in Kirindy Forest (western Madagascar) and compared them with sympatric and longer living *F.* cf. *nicosiai*. Moreover, we included data from wild *F. labordi* that were singly housed under ambient conditions with daily food and water supply. Gastrointestinal parasite prevalence of wild *F. labordi* increased dramatically during the last 3 months of their lives, which include the reproductive period. *Furcifer* cf. *nicosiai* was found to have a belated increase in gastrointestinal parasites compared to *F. labordi*. In *F*. cf. *nicosiai* higher prevalence of blood parasites were found, which probably result from the longer exposure to the arthropod intermediate host. Both species showed infestations with ectoparasites, which peaked in the rainy season but disappeared towards the dry season. Male *F. labordi* showed a significantly higher prevalence of gastrointestinal - and ectoparasites and higher intensities of coccidians and ectoparasites than females. Males of *F.* cf. *nicosiai* exhibited higher prevalence of blood- and ectoparasites, as well as higher intensities in ectoparasites. Caged individuals of both sexes showed delayed senescence, reduced parasite burden and lived longer than their wild conspecifics. Overall, the increase in the prevalence in gastrointestinal - and blood parasites towards the disappearance of the wild population of *F. labordi* indicates that this species invests comparatively less energy in efficient immune system function, supporting the prediction of life history theory.

## Highlights

• Wild *F. labordi* show an increase of parasites towards their die-off
• Male *F. labordi* exhibit higher parasite burden than longer living females
• Longer living *F.* cf. *nicosiai* show belated increase in parasite burden
• Captive *F. labordi* were less infected and longer living than wild specimens

## Graphical abstract

**Figure:**
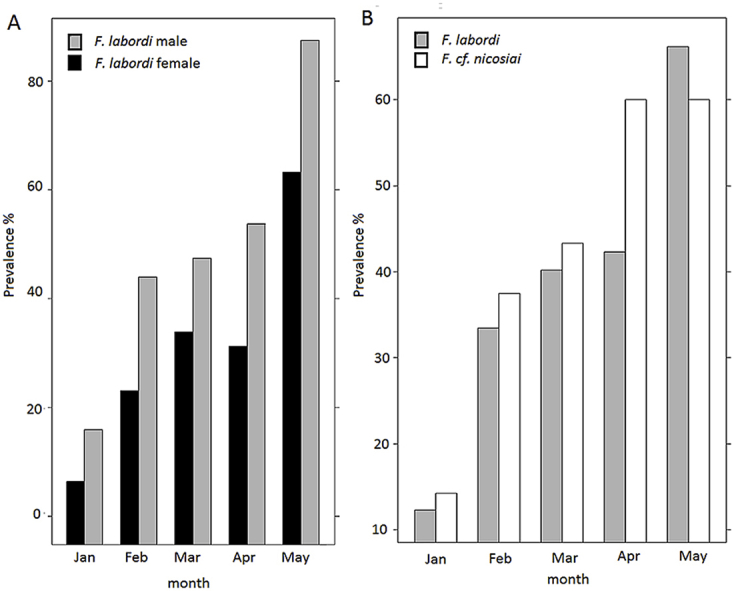

## 1 Introduction

During aging, the accumulation of molecular and cellular damage is thought to lead to functional decline, resulting in compromised health and, finally, death (Kirkwood, 2005). According to “disposable soma theory”, natural selection evaluates how much an organism invests into growth and reproduction *versus* self-maintenance and repair, and hence, determines the rate of aging and lifespan (Kirkwood, 1977; Kirkwood and Holliday, 1979). Regarding immunity, “fast-living” species are supposed to rely more on low-cost nonspecific and inflammatory immune defenses, whereas “slow-living” species exhibit rather cost-intensive stronger specific and especially antibody-mediated immunity, which is required as defense against parasites, for instance (Lee, 2006). Besides these trade-offs, within vertebrates the functioning of the immune system changes over time, from the development of adaptive immunity at birth to the deterioration of the system at old age (Albright and Albright, 1994; Malaguanera et al. 2001; Humphreys and Grencis, 2000; Hayward, 2013). The latter process is known as immunosenescence, which is characterized by a down-regulation of type Th2 immunity, which is involved in parasite resistance (Malaguanera et al. 2001). For example, tissue destruction is often caused by parasites and Th2 cell mediated immunity evolved as an adaptive tissue repair mechanism that quickly heals the wounds they inflict (Allen and Wynn, 2011).

Because non-invasive measurements of immunocompetence in free-ranging animals are challenging, parasite burden is often used as a surrogate index of general health (Zuk, 2002; Hämäläinen et al., 2015). Especially gastrointestinal parasites can be monitored with marginally invasive means via fecal egg counts. While many parasites induce only moderate clinical symptoms, they may provoke considerable energetic costs due to immune defense investment required to countervail the effects of infection (Zuk et al., 1996; Marcogliese and Pietrock, 2011). These costs are intensified by reduced health due to poor nutrition Marcogliese and Pietrock (2011) or other stressors (Zuk et al., 1996), resulting in a trade-off between parasite resistance and reproductive performance (Helle et al., 2004; Mills et al., 2010) and an accelerated rate of immunosenescence (Hudson et al., 1992). Furthermore, parasite infections induce additional costs, including enhanced risks of predation (Temple, 1987; Graham, 2008) and further infections by additional parasites (Petney and Andrews, 1998; Cox, 2001; Bordes and Morand, 2009; Johnson and Buller, 2010), resulting in a more than linear increase of the associated costs (Ezeamama et al., 2008).

Host sex is one of the important determinants of the immune function profile (Alexander and Stimson, 1998). Among mammals, a male bias in parasite infection rates is common (Moore and Wilson, 2002). Ultimately, sex differences in immune responses are thought to originate from sex-specific life history strategies, where males benefit from investing into reproductive effort during their prime reproductive age, whereas female fitness is generally improved by a longer reproductive lifespan due to their higher investment in each offspring. These conditions can favor greater female investment into health maintenance, which ought to prolong their lifespan, whereas males are more likely to invest in competitive success (Williams, 1957; Clutten-Brock and Isvaran, 2007), resulting in comparatively accelerated immunosenescence. For example, when male greater kudu *Tragelaphus strepsiceros* reach adulthood and begin to rut successfully, they compete so intensely in the annual rut that they commonly either die from exhaustion or are killed by predators (Owen-Smith, 1993).

Labord's chameleon (*Furcifer labordi*) from the highly seasonal deciduous dry forests in western and southwestern Madagascar has a post-hatching lifespan of only 4–9 months (Karsten et al., 2008; Eckhardt et al., 2017). This extreme life history makes this species an interesting model to study potential mechanisms of accelerated senescence, especially because longer-lived sympatric congeners are available for comparative studies. During their short lives, this species undergoes hatching, juvenile growth, maturation and courtship followed by death of both sexes early during the annual dry season (Karsten et al., 2008; Eckhardt et al., 2017). Females tend to enjoy a slight longevity advantage, whereas no significant intersexual differences in lifespan were found in caged individuals that were kept under ambient conditions (Eckhardt et al., 2017). With such a fast life history, chronic physiological stress might proximately contribute to immune suppression, which in turn facilitates parasite infections and ultimately leads to death. These mechanisms have been demonstrated in semelparous marsupials (Bradley et al., 1980; Lee et al., 1982; Dickman and Braithwaite, 1992), where males in wild populations died considerably earlier compared to females, whereas captive males outlived their wild conspecifics.

To investigate to which extent changes in parasite burden across the lifespan contribute to the early die-off in *F. labordi*, we examined their gastrointestinal-, blood- and ectoparasite burden. To this end, we determined the prevalence of ectoparasites as well as gastrointestinal parasite reproductive stages (e.g. eggs, oocysts), and blood parasites (e.g. microfilariae) encountered in fecal and blood samples obtained from *F. labordi* throughout their life. Our study included two comparisons; one between wild *F. labordi* and their sympatric and longer-lived congener *F.* cf. *nicosiai*, and one with *F. labordi* kept in single cages under ambient conditions, which buffered them substantially from physiological stress and to some degree from parasite infection. We predicted an increase in parasite loads towards the end of the reproductive season in wild *F. labordi* and a reduced increase in *F.* cf. *nicosiai*. Furthermore, as age-related changes in immunocompetence should be delayed in the longer-lived females, we predicted female *F. labordi* to exhibit a lower and comparatively slower increase in parasite load than males. Additionally, caged *F. labordi,* which were safeguarded against extrinsic mortality, the costs of reproduction and starvation, were expected to exhibit slower rates of aging, and hence reduced parasite infection, compared to their wild conspecifics.

## 2 Materials & methods

### 2.1 Study site, study species and capture-mark-recapture

The study was conducted at Kirindy Forest (44°39′E, 20°03′S, 30–60 m asl), one of the largest remaining tracts of dry deciduous forests in central western Madagascar. The local climate is characterized by a hot rainy season from November until March and a cool dry season from April until October. The forest is relatively dense and has undergone selective logging (Kappeler and Fichtel, 2012).

*Furcifer labordi* is a medium-sized and sexually highly dimorphic chameleon from the western and southwestern regions of Madagascar (Glaw and Vences, 2007). Males have a body size of approx. 100 mm, and females have a body size of 73 mm. *Furcifer* cf. *nicosiai* is a relatively larger species, also sexually dimorphic, and appears to be associated with intact dry forests (Jesu, Mattioli and Schimmenti, 1999; Glaw and Vences, 2007). Males reach a body size of 136 mm and females 102 mm (Eckhardt et al., 2019). Concerning ecological studies in the Kirindy forest, both species differ significantly in point of hatching, growth rates and roosting heights, which might suggest some interspecific niche segregation (Eckhardt et al., 2019).

Chameleons were located at night using flashlights. The roost perch of each detected chameleon was marked with flagging tape. Collected animals were placed in a cloth bag and handled the following morning. Snout-vent length and body mass were recorded, as well as age and sex. Animals were released at their point of capture within 12 h. Sampling took place over three field seasons: November 2013–July 2014, and January 2015–July 2015, and October 2015–December 2015.

### 2.2 Experimental housing

We collected a total of 20 male and 20 female juveniles of *F. labordi* in early January, at approximately two months of age. On January 2014 as well as 2015, each 10 males and 10 females were collected. They were kept individually without visual contact in cylindrical outdoor enclosures (90 cm height, 60 cm diameter) made of nylon screen. The enclosures were equipped with branches and artificial plants. In order to experience the same temperature fluctuations and daylight conditions as their wild conspecifics, caged animals were positioned in a large outdoor cage in the forest. Chameleons received a standardized amount of food (crickets, grasshoppers or butterflies), adjusted to their age and size to match growth and final size of the wild population. Water was offered daily with a spray flask. We used the Kaplan – Meier estimator to assess the survival probability of both sexes in captivity.

### 2.3 Analyses of gastrointestinal parasites

Fecal samples were collected opportunistically from cloth bags or during animal handling and stored in 70% ethanol. Parasite identification was based on size, shape and internal structure of eggs, oocysts and larval stages. We determined prevalence, intensity of egg and/or oocyst shedding and morphospecies richness of helminth and protozoan parasites. Egg shedding intensity was estimated using fecal egg counts (FEC/g feces) with a modified McMaster flotation egg counting technique (Sloss et al., 1994), a method commonly employed to estimate shedding intensity in wild populations of lizards (Hallas and Bull, 2006; Fenner et al., 2011). Fecal samples that weighed less than 0.1 g, were directly dispersed with a toothpick in a counting chamber, subsequently diluted with water and examined. Although FECs are a generally used method to study parasite infections, the method has been criticized for its potential inaccuracy, as parasite egg shedding rates fluctuates over time and a sample may not always contain the eggs of a parasite that is present in the host (Hallas and Bull, 2006). However, as coccidians are considered as harmful protozoans (Modrý et al., 2000; Schneller et al., 2008), the number of their spores (oocysts) reflects the degree of intestinal cells that are infected with macrogametes. Therefore, we evaluated the number of oocytes in the fecal samples.

### 2.4 Analyses of blood parasites

We quantified the prevalence of blood parasites such as microfilaria. Blood-sucking arthropods serve as intermediate hosts and infective microfilarial stages are transmitted to other reptiles (Mancianti et al., 2000). False-negative results are rare because both the adults and the microfilariae of the members of the family Onchocercidae are long-lived, and several species often produce significant microfilaremia (Széll et al., 2001). For detection of filariid infections, we used blood smears (Irizarry-Rovira et al., 2002). Here, a drop of blood was taken by lateral puncture of the caudal vein and placed on a microscope glass slide and distributed applying a second slid. After air-drying, blood smears were processed with a rapid differential haematology staining, using the Diff-Quik staining solution system (Medion Diagnostics AG, Düdingen, Switzerland). Samples were analyzed for prevalence of blood parasites applying a brightfield microscope (Zeiss Primo Star) and 100-fold or 400-fold magnification. No samples were taken from individuals that weighted less than 5 g or females that were very close to oviposition*.*

### 2.5 Analysis of ectoparasites

The body of each chameleon was inspected with a magnifying lens to identify and count the number of ectoparasites.

### 2.6 Statistical analyses

Generalized linear mixed effects (GLMM) models for longitudinal data were used to model the parasite data. As fixed factors, we added month (age), sex and species, while ID was included as a random factor for recaptured individuals. We conducted models including both species and models including *F. labordi* only. To test effects on prevalence, we used a binominal distribution and for intensity, we used a Poisson distribution (e.g. Peterson and Lello, 2003; Verbeke and Molenberghs, 2005). For all models, we compared the respective full model with the null model by using a likelihood ratio test. We also checked for model stability by determining Variance Inflation Factors (VIF) for a standard linear model excluding the random effects. In addition, we visually inspected normality and homoscedasticity with residual plots. For model analysis, we used the package lme4 (Bates et al., 2014). All data analysis was conducted in R (R-Code Team, 2017). To test for interspecific differences according to multiple infections with different endoparasite taxa, we conducted a two-proportion Z – test.

## 3 Results

We observed first hatchlings of *F. labordi* in mid-October, at the onset of the rainy season. This cohort grew up and reached maturity not later than February. Towards the end of the mating season, males disappeared in late May, whereas females were found until the beginning of July. Thus, the lifespan of animals in this population ranged from six to nine month. However, after a remarkably long rainy season, males and females survived considerably longer, one adult female even survived until the next breeding season (Eckhardt et al., 2017). In November, we found a cohort of juvenile *F.* cf. *nicosiai* that had hatched during the previous active season. These juveniles grew up to adult size by February, i.e. more slowly than juvenile *F. labordi* [see also Eckhardt et al. (2019)]. Hatchlings were detected around mid-February. Adult males were encountered until mid-June, and adult females until the end of June. After this date, we only detected small juveniles that ceased growing. However, after an unusually long rainy season adult males and females were found for longer and we found two adult females that overwintered the previous dry season. Concerning the 40 caged individuals of *F. labordi*, we found no significant differences in survival probability between males and females. Median lifespan for females was 9.5 months and for males 8.2 months. Maximum lifespan for females was 11.5 months and for males 16 months [see also Eckhardt et al. (2017)].

In the coproscopic analyses, we identified one protozoan morphotype that was assigned to the Coccidians (*Isospora* sp.). We also detected two egg morphotypes that were assigned to the Cestoda (Cyclophyllidae, *Oochoristica* sp.) and Nematoda belonging to the family Heterakidae (*Spinicauda* sp.). Additionally, we found Oxyurids in five samples and Ascarids (*Hexametra* sp*.*) in two samples. Due to their rare occurrence and the possibility that the latter two nematode taxa were parasites of prey species, we exclude them from our analysis.

**Fig. 1:**
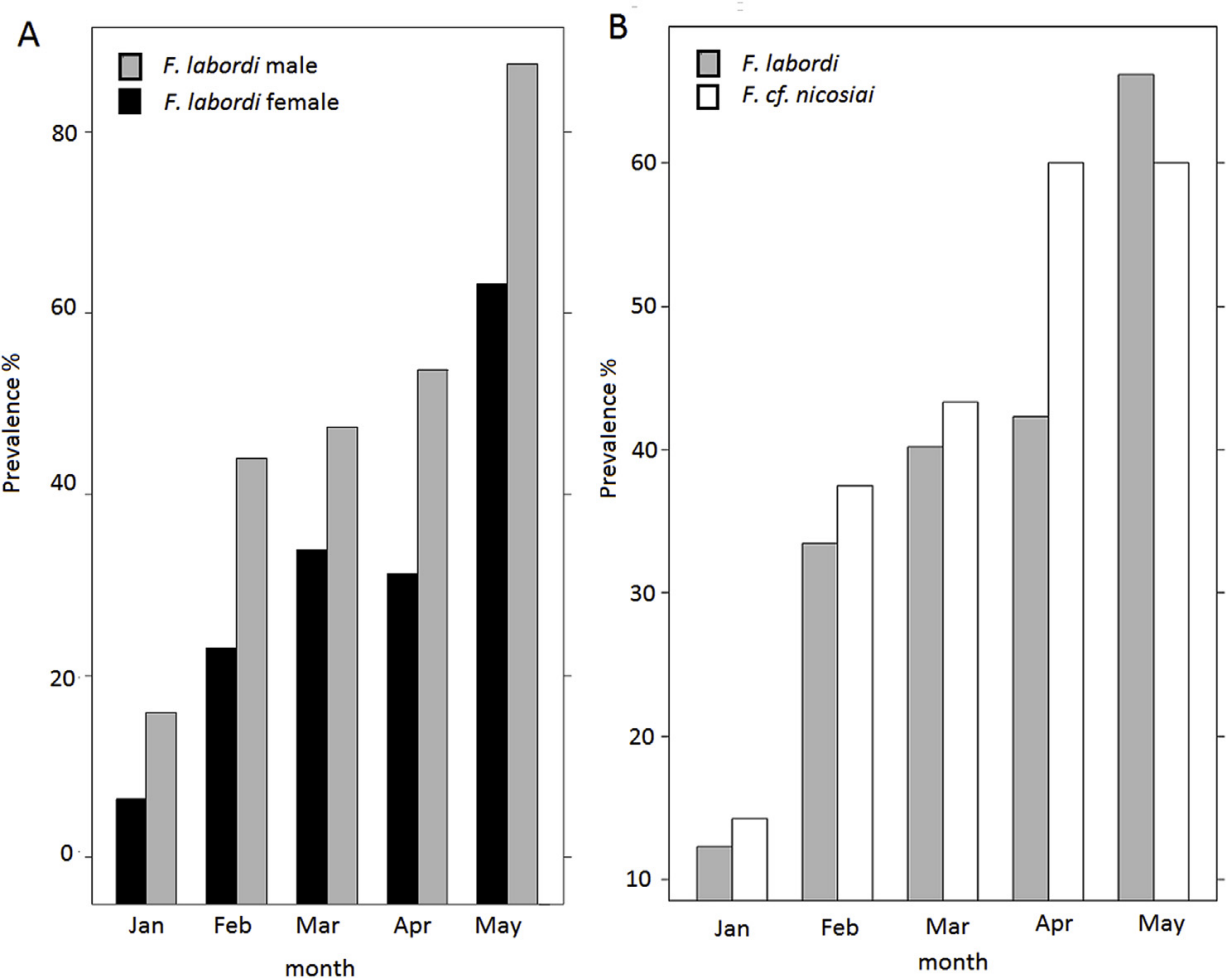
Prevalence in percentage of gastrointestinal parasitic infection A) in *F. labordi* males and females, B) *F. labordi* and *F.* cf. *nicosiai* (adult stages).

Between hatching in mid-October until December, we did not detect any infestation in the fecal samples of *F. labordi*. However, from January onwards, the prevalence of gastrointestinal parasites in this species increased significantly from 12.3% in January until 57.1% in June (z = −8.539; P < 0.001, Fig. 1A, Table 1 and Table 4 A). Moreover, adult males showed a significantly higher prevalence compared to females (z = 4.432; P < 0.001, see Fig. 1A and Table 4 A). In *F.* cf. *nicosiai*, we found a low prevalence (6.8%, n = 207) of gastrointestinal parasites in fecal samples of hatchlings and juveniles that were sampled between mid-February and mid-July. From January onwards, we also detected an increase of the prevalence of gastrointestinal parasites, in this species from 14.3 to 60% in May. When comparing gastrointestinal parasite prevalence among adult *F. labordi* and *F*. cf. *nicosiai*, we found that prevalence was higher in the latter species (z = −9.211; P < 0.001, see Fig. 1B and Table 4B). In contrast to *F. labordi*, the prevalence of gastrointestinal parasite infection was lower in male *F.* cf. *nicosiai.* Regarding the shedding intensity of *Isospora* sp., we detected an average number of oocysts per g feces (OPG)/month in *F. labordi* ranging between 2600 and 73747. The highest number of oocysts was detected in May. Comparing both species, we did not find any significant differences. However, in male *F. labordi*, we detected a highly significant increase of oocyte number in May (Table 5).

**Table 1:** Number of fecal samples collected per species (*F. labordi* and *F.* cf. *nicosiai*) and sex (*F. labordi*) per month.

Species/sex	Jan	Feb	Mar	Apr	May	Jun
*F. labordi*	204	183	224	164	64	8
*F.* cf. *nicosiai*	27	24	30	17	10	9
*F. labordi male*	126	92	114	41	8	0
*F. labordi female*	78	99	128	133	56	8

**Fig. 2:**
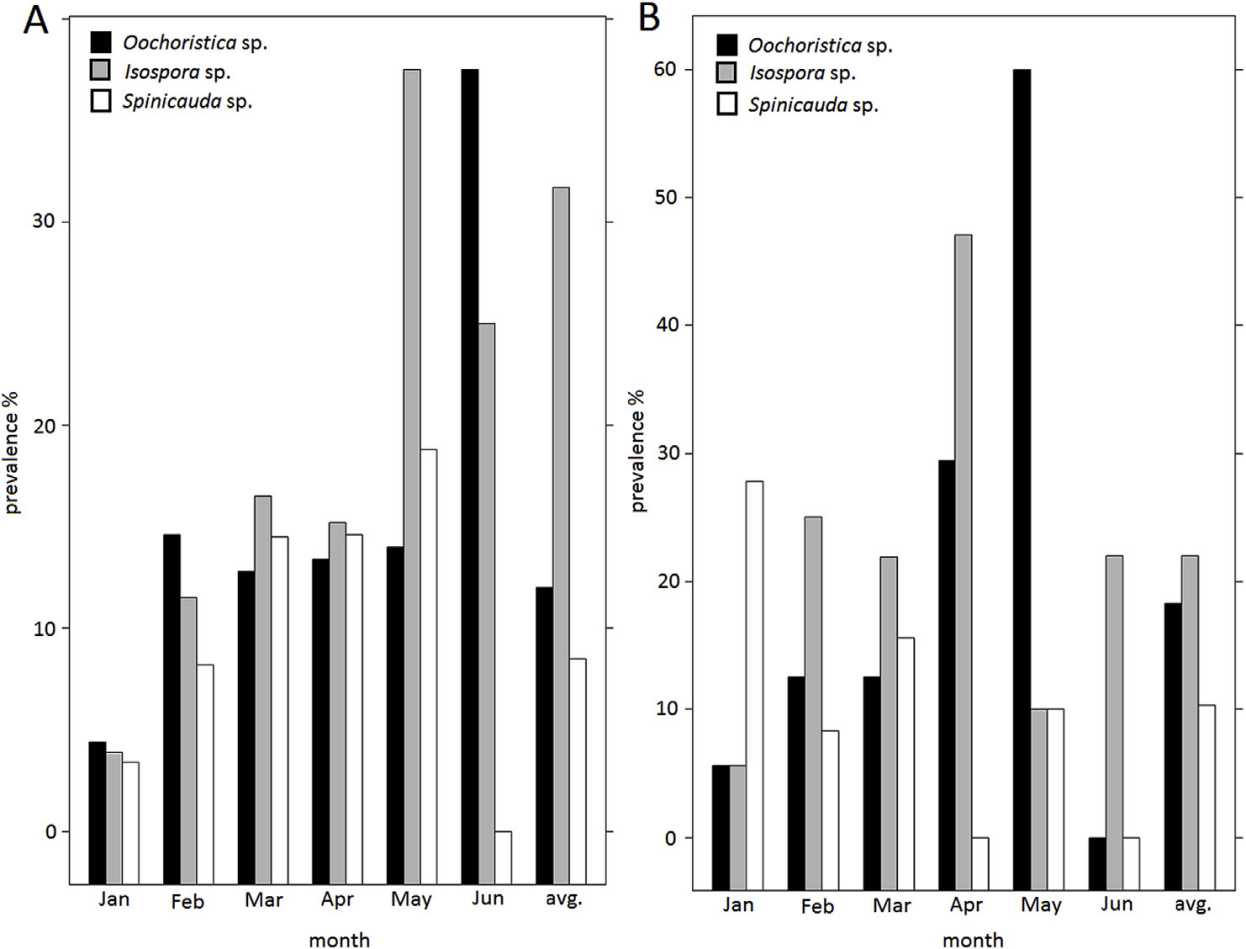
Composition of gastrointestinal parasite taxa in the fecal samples of A) adult *F. labordi* and B) adult *F.* cf. *nicosiai* from January to June and in total.

Concerning the taxonomic composition of gastrointestinal parasite taxa in the fecal samples of *F. labordi*, *Isospora* sp. (Eimeriidae, Coccidia) were most common and present in 31.7% of all fecal samples. *Oocherisitica* sp. (Cyclophyllidae, Cestoda) (12%) and *Spinicauda* sp. (Heterakidae, Ascaridida) (8.5%) had lower prevalences. In *F.* cf. *nicosiai*, we found a similar parasite composition, with 22% *Isospora* ssp. 18.3% *Oocheristica* sp. and 10.3% *Spinicauda* sp. prevalence (Fig. 2).

Regarding multiple infections, the number of gastrointestinal parasite taxa ranged from 1 to 3 in infected samples. Of the 381 infected samples, 7.9% contained two gastrointestinal parasite taxa. Three morphotypes where only found in 0.5% of the infected samples. Double infection rates for males and females were 12.1% and 8.1%, respectively. Triple infections where only found in 1.3% of female samples. As the rate of multiple infections was rather low, no clear dynamics with age could be observed. Of the 30 double infections, 53.3% contained *Spinicauda* sp. and *Oocheristica* sp., 30% contained *Isospora* sp. and *Ooceristica* sp., and 16.7% contained *Isospora* sp. and *Spinicauda* sp. In 118 infected samples of *F*. cf. *nicosiai*, we found 7 double infections (5.9%). The two-proportion Z-test revealed no significant differences of the rate of double infections between both species (χ^2^ = 0.252, df = 1, P = 0.615).

Among the caged chameleons*,* two males exhibited coccidiosis from February onwards and subsequently died at an age of approximately five months. The number of oocytes/g in their samples fluctuated between 2 and 86750, with an average increase towards the end of their lives. In parallel with the increase in oocytes, their body mass decreased dramatically. Additionally, we found that two males and two females were infected with *Spinicauda* sp.

**Fig. 3:**
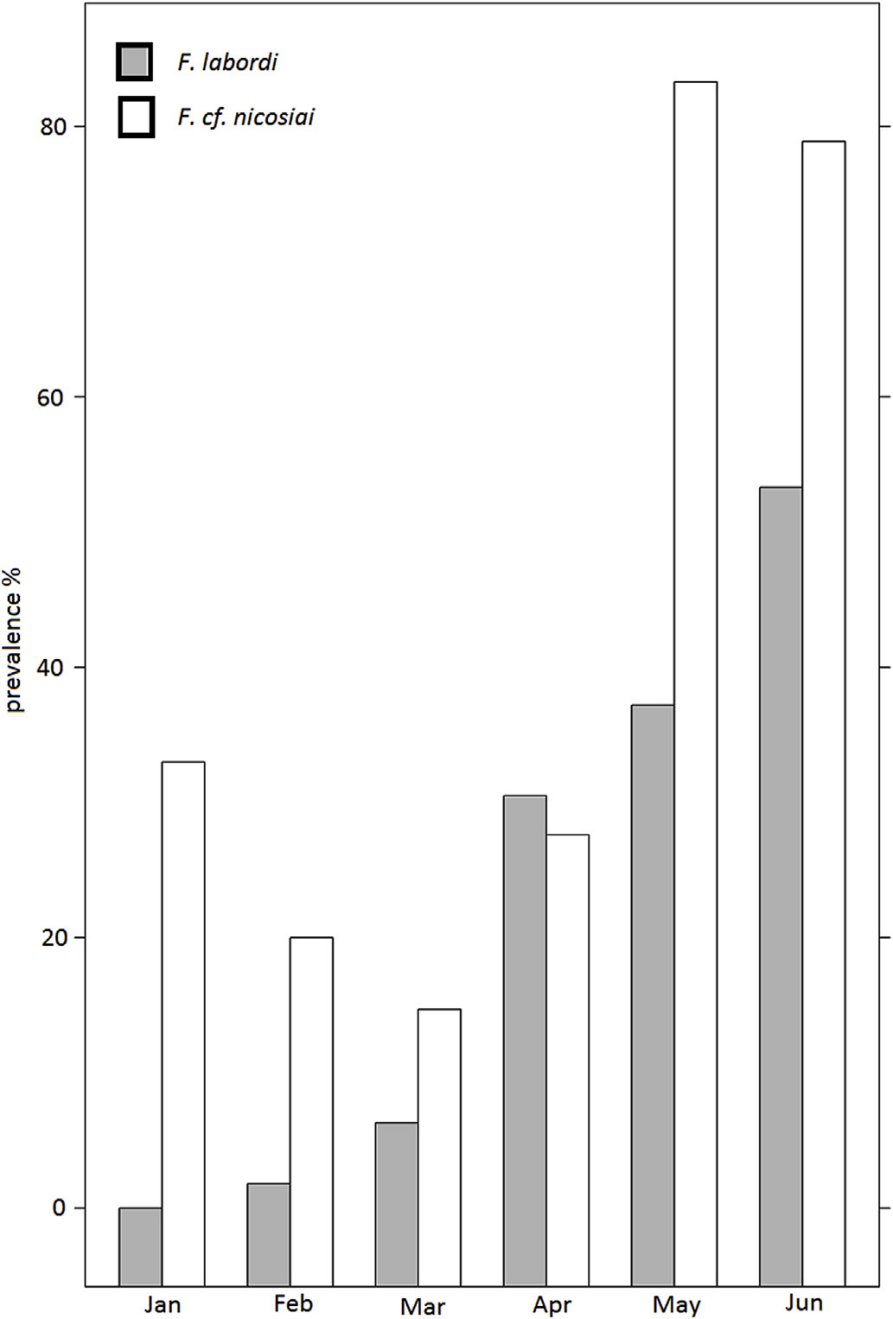
Prevalence of filarial infection in *F. labordi* and *F.* cf. *nicosiai.*

The analysis of blood samples revealed that both species were infected by the nematode species *Foleyella* aff. *furcata*. Parasites of this genus have a limited geographic distribution and have been found only in the lizard family Agamidae and Chamaeleonidae (Bartlett, 1986). *Foleyella* spp. are long-lived and viviparous (Brygoo, 1963). Adults are relatively large and predominantly inhabit muscle or skin whereas their progeny, the so-called microfilaria circulate in the blood of the host (Fenner et al., 2011). The prepatent period takes about six months (Széll et al., 2001). In *F. labordi*, the prevalence of filarial infection increased from the beginning of the reproductive season (Fig. 3, Table 2). Within *F*. cf. *nicosiai*, incipiently the prevalence of blood parasites decreased from January until March, but hereafter increased remarkably from April toward June. The interspecific comparison revealed that *F.* cf. *nicosiai* was more frequently infected by *Foleyella* aff. *furcata* than *F. labordi* (z = 2.187; P < 0.05)*.* Concerning intersexual differences of prevalence in *F.* cf. *nicosiai*, we found that males showed higher significant prevalence of filarial infection. (z = 2.34; P < 0.05, see Fig. 3 and Table 4C). Among the chameleons in the cages, we detected filarial infection in one male.

**Table 2:** Number of blood samples collected per species per month.

Species	Jan	Feb	Mar	Apr	May	Jun
*F. labordi*	6	109	94	59	44	13
*F.* cf*. nicosiai*	3	15	32	30	13	11

**Fig. 4:**
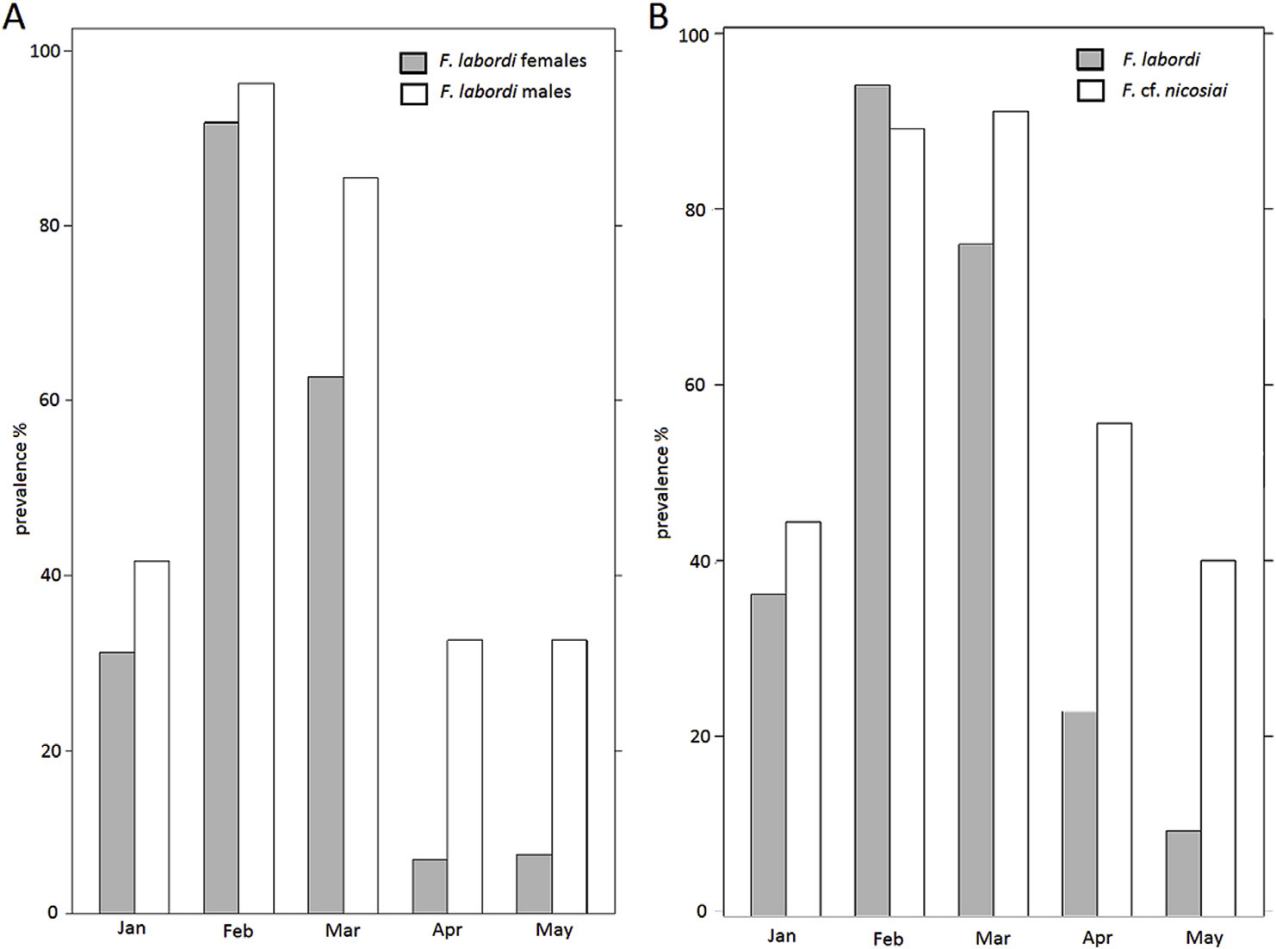
Prevalence of mite infestation in A) *F. labordi* males and females, B) *F. labordi* and *F.* cf. *nicosiai.*

**Fig. 5:**
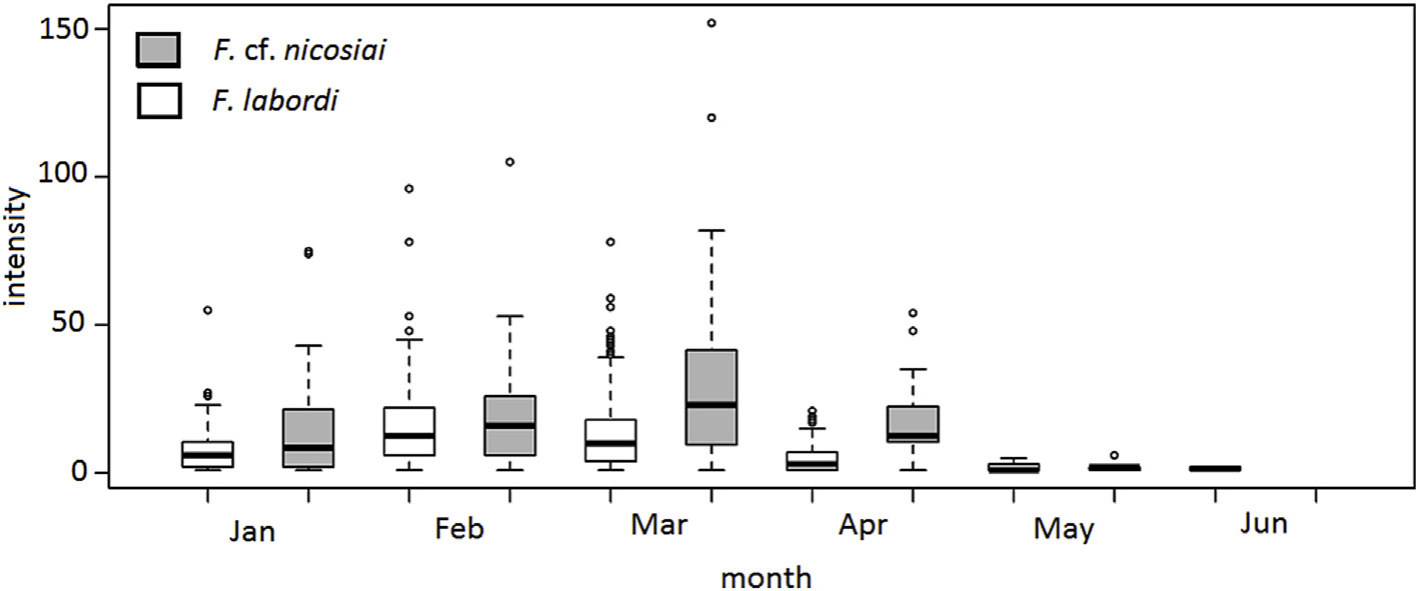
Intensity of mite infestation in adult *F. labordi* and *F.* cf. *nicosiai*.

We identified acarians of the family Trombiculidae (trombiculids) as ectoparasites in both *Furcifer* species, which were mostly located in the axillary pits. Acarians were detected from January onward. Their prevalence peaked in February and March and they were no longer detectable in June. Comparing both species, we found that a higher prevalence of acarians in *F.* cf. *nicosiai* (see Fig. 4, Table 3 and Table 4 D). Moreover, we found that the number of trombiculids was significantly higher in *F*. cf. *nicosiai* compared to *F. labordi*. Males of both species exhibited a higher prevalence and intensity of these ectoparasites than females (z = 5.617; P < 0.001). In *F. labordi,* males showed a prevalence of 66.7% (n = 486) and carried 16.6 ± 15.1 mites, whereas 48.5% of the females were infested with an average of 8.0 ± 8.1 mites. In *F*. cf. *nicosiai*, 82.3% of all examined males (n = 96) were infested and showed 31 ± 27 mites, whereas 57.6% of females (n = 92) were infested and carried on average 12 ± 12.7 mites. Regarding intensity of acarian infestation, we did not detect significant interspecific differences, though specimens of *F*. cf. *nicosiai* tended to have more mites compared to *F. labordi* (see Fig. 5, Table 6). Similar to the prevalence, the intensity increased at the peak of the rainy season and decreased toward the dry season (see Fig. 5, Table 6). Among the caged animals, we rarely detected acarians and therefore excluded them from any statistical analysis.

**Table 3:** Number of individuals per species/sex and month that were inspected for ectoparasites.

Species/sex	Jan	Feb	Mar	Apr	May
*F. labordi*	207	205	308	246	119
*F.* cf. *nicosiai*	52	37	55	36	17
*F. labordi male*	127	108	166	86	20
*F. labordi female*	80	97	142	160	99

**Table 4:** Predictors of prevalences of A) gastrointestinal parasites in *F. labordi* B) gastrointestinal parasites of both species*,* C) blood parasites in both species, D) ectoparasites in both species.

A) Prevalence: gastrointestinal parasites *F. labordi*							
Parameter estimates					Likelihood ratio test		
Fixed effects	Est.	SE	z	P	χ²	df	P
Intercept	−2.7265	0.3193	−8.539	**<0.001**	110.75	6	**<0.001**
Sex (male)	0.8484	0.1914	4.432	**<0.001**			
Feb	1.5103	0.3007	5.023	**<0.001**			
Mar	1.8755	0.2986	6.280	**<0.001**			
Apr	2.1040	0.3263	6.448	**<0.001**			
May	3.3748	0.4497	7.505	**<0.001**			
Jun	2.7370	0.8500	3.220	**<0.01**			
**B) Prevalence: gastrointestinal parasites *F. labordi* and *F.* cf. *nicosiai***							
**Parameter estimates**					**Likelihood ratio test**		
**Fixed effects**	**Est.**	**SE**	**z**	**P**	**χ**^**2**^	**df**	**P**
Intercept	−2.6471	0.2874	−9.211	**<0.001**	129.93	8	**<0.001**
*F*. cf. *nicosiai*	1.0532	0.3197	3.295	**<0.001**			
Sex (male)	0.8159	0.1832	4.453	**<0.001**			
Feb	1.4755	0.2726	5.413	**<0.001**			
Mar	1.8089	0.2689	6.727	**<0.001**			
Apr	2.0870	0.2974	7.018	**<0.001**			
May	3.2360	0.4002	8.085	**<0.001**			
Jun	2.0317	0.6291	3.230	**<0.01**			
*F*. cf. *nicosiai* (male)	−2.0865	0.5175	−4.032	**<0.001**			
**C) Prevalence: blood parasites *F. labordi* and *F.* cf. *nicosiai***							
**Parameter estimates**					**Likelihood ratio test**		
**Fixed effects**	**Est.**	**SE**	**z**	**P**	**χ**^**2**^	**df**	**P**
Intercept	−2.7623	0.7641	−3.615	**<0.001**	90.78	6	**<0.001**
*F.* cf. *nicosiai*	0.7187	0.3287	2.187	**<0.05**			
Sex (male)	0.7567	0.3233	2.340	**<0.05**			
Feb	−1.7501	1.0150	−1.724	0.08468			
Mar	−0.3750	0.7466	−0.502	0.61550			
Apr	1.1826	0.7179	1.647	0.09950			
May	2.3222	0.7508	3.093	**<0.01**			
**D) Prevalence: ectoparasites *F. labordi* and *F.* cf. *nicosiai***							
**Parameter estimates**					**Likelihood ratio test**		
**Fixed effects**	**Est.**	**SE**	**z**	**p**	**χ**^**2**^	**df**	**P**
Intercept	−1.3332	0.2198	−6.066	**<0.001**	607.48	7	**<0.001**
Sex (male)	0.9934	0.1769	5.617	**<0.001**			
*F.* cf. *nicosiai*	0.8087	0.2213	3.654	**<0.001**			
Feb	3.6267	0.4200	8.634	**<0.001**			
Mar	2.1469	0.3087	6.955	**<0.001**			
Apr	−0.3134	0.2158	−1.452	0.14649			
May	−1.3439	0.3434	−3.914	**<0.001**			
Jun	−2.7206	0.7833	−3.473	**<0.001**

**Table 5:** Shedding intensity of coccidian oocysts of *F. labordi* and *F.* cf. *nicosiai*.

Intensity: *Isospora* sp. of *F. labordi* and *F*. cf. *nicosiai*								
Parameter estimates						Likelihood ratio test		
Fixed effects	Est.	SE	df	t-value	p	χ^2^	df	P
Intercept	17358.7	20290.8	192.0	0.855	0.393	29.39	8	**<0.001**
Mar	−13592.0	24402.5	189.9	−0.557	0.578			
Apr	−2188.9	25262.6	197.2	−0.087	0.931			
May	21675.3	25424.7	195.0	0.853	0.395			
Sex (male)	−14996.7	29319.3	192.0	−0.511	0.610			
*F*. cf. *nicosiai*	−10617.4	19035.0	192.1	−0.558	0.578			
Mar (male)	14460.4	37649.4	191.1	0.384	0.701			
Apr (male)	5659.0	43324.8	193.6	0.131	0.896			
May (male)	388477.9	84369.7	192.2	4.604	**<0.001**

**Table 6:** Intensity of ectoparasites of *F. labordi* and *F.* cf. *nicosiai*.

Intensity: ectoparasite infestation of *F. labordi* and *F.* cf. *nicosiai*							
Parameter estimates					Likelihood ratio test		
Fixed effects	Est.	SE	t-value	P	χ^2^	df	P
Intercept	1.775	0.139	12.808	**< 0.001**	290.43	15	**<0.001**
*F*. cf. *nicosiai*	0.402	0.205	1.959	0.0501			
Feb	0.179	0.144	1.248	0.212			
Mar	−0.119	0.161	−0.739	0.46			
Apr	−1.107	0.228	−4.861	**< 0.001**			
May	−1.508	0.398	−3.789	**< 0.001**			
Jun	−1.547	0.834	−1.854	0.064			
Sex (male)	0.076	0.175	0.436	0.663			
*F.* cf. *nicosiai* Feb	−0.232	0.247	−0.939	0.348			
*F.* cf. *nicosiai* Mar	0.333	0.241	1.380	0.167			
*F.* cf. *nicosiai* Apr	0.899	0.294	3.061	**< 0.01**			
*F.* cf. *nicosiai* May	−0.286	0.627	−0.456	0.648			
Male Feb	0.675	0.193	3.506	**< 0.001**			

## 4 Discussion

### 4.1 *Furcifer labordi* in nature

As predicted, among wild living specimens of *F. labordi*, we detected a massive increase of gastrointestinal parasites related to prevalence and mixed infections over the reproductive period towards the dry season. A similar pattern of decline in immune function was reported for feral Soay sheep *Ovis aries* (Hayward et al., 2009). In contrast, Hämäläinen et al. (2015) found that parasite prevalence and morphotype richness decreased with increasing age in a small and relatively short-lived primate species (*Microcebus murinus*), indicating acquired immunity by older specimens. Besides, studies focusing on parasite infection with regard to aging in the wild are lacking. However, the detected increase of prevalence of gastrointestinal parasites according to time is probably linked to a decrease in immune functioning in *F. labordi*. We also found that males, but not females, of *F. labordi* showed a remarkable increase of oocyst shedding towards the end of the reproductive season. Concerning the prevalence of coccidian oocysts in the fecal samples, Modrý et al. (2000) found that 32.5% of 83 examined chameleon specimens from east Africa were infected with coccidians, which is similar to our findings in both species. Additionally, in a coproscopic study on chameleons in captivity, Biallas (2013) found that *Isospora* was regularly detected (21.7%). Accordingly, coccidians in general seem to be frequent gastrointestinal parasites in both, wild living and caged chameleons. *Isospora* sp. was the most commonly detected gastrointestinal parasite taxon in our study, but we may not have detected all kinds of gastrointestinal parasites, such as trematode eggs, that are too heavy to float during the flotation process in saturated NaCl solution. In their study, Morsey et al. (2012) found that 26.1% of 115 specimens of the common chameleon, *Chamaeleo chamaeleon,* were naturally infected with the digenetic trematode *Postorchigenes* sp. and 32.1% with *Malagashitrema* sp. Thus, the number of gastrointestinal parasite taxa and their prevalence might be underestimated in our study. Moreover, as the samples were stored in ethanol, a detection of intestinal flagellates and ciliates was not possible. Although these groups are mostly considered as commensals, under physiological stress, they can have a severe effect on the host (Schneller et al., 2008).

Regarding blood parasites, we detected filarias belonging to the genus *Foleyella*. Here, we detected an increase of prevalence towards the dry season, which might have a severe influence on the individual's health. However, little is known about the clinical signs of foleyellosis in chameleons. Higher mortality rates were noted in *Foleyella*-infected chameleons than in uninfected animals, which were transported from a tropical to a temperate zone (Brygoo, 1963). In their study, Maia et al. (2014) report a relatively high incidence of filarial infections in the Malagasy chameleon genus *Furcifer*, which we reported as well.

Concerning ectoparasites, we first detected acarian infestation in January, when humidity was relatively high. Their prevalence was highest in February and March, but decreased towards the dry season, eventually decreasing to zero. Thus, their detection seems to be highly associated with the rainy season, when chameleons are still in good physical conditions. In their study of the impact of tick load on the fitness of their lizard hosts, Bull and Burzacott (1993) did not find any influence on the longevity of the sleepy lizard *Tiliqua rugosa*. In addition, we found that trombiculids were obviously restricted to axillary situated so called “mite pockets”.

At first sight, mite pockets are paradoxical structures as they seem to provide an optimal environment for the mites, giving protection from solar radiation, high temperatures, desiccation, and offering easily penetrated skin. Here, the most likely hypothesis for their function is that they reduce damaging effects of mite infestations (Arnold, 1986). Mites are probably attracted to the pockets because they provide ideal conditions, whereas in return these invaginations appear to ameliorate much of their potential damage. These pockets have a large internal area of exposed skin compared with their volume, which enables large numbers of mites to be concentrated in places where they do not interfere with general cutaneous function. In particular, the epidermis is resilient and recovers rapidly after a mite has fed. The shape of the pocket enables large numbers of lymphoid cells to be concentrated around the feeding mites and it is probable that these cells reduce the effects of antigens and any pathogens introduced by the feeding mites, as well as contributing to their diet. Thus, due to the peak of the infestation in the rainy season and evolutionary adaptation to these parasites, we do not consider acarian infestation as a decisive factor that is influenced by immunosenescence. However, these parasites cause blood loss and are potential vectors for pathogens that can have negative impacts on the health of the host (Schneller et al., 2008).

### 4.2 Interspecific comparison of *F. labordi* and *F.* cf. *nicosiai*

We found higher prevalence of gastrointestinal parasites in *F*. cf. *nicosiai*, but we found the first infections in *F. labordi* approx. 2–3 months after hatching. Among juvenile *F*. cf. *nicosiai,* which hatched around mid-February, we rarely detected any gastrointestinal parasites until the dry season in June. The delayed occurrence of gastrointestinal parasite infection in *F.* cf. *nicosiai* might be caused by a higher energy investment in the immune system and especially in parasite defense. In contrast to *F. labordi*, juveniles of this species exhibit rather slow growth rates, later sexual maturity and higher rates of recaptures and therefore potentially higher probability of survival (Eckhardt et al., 2019) that might enable them to invest comparatively more energy into immune defense. Besides slow growth rates, juveniles probably digest less food insects and are therefore less prone to gastrointestinal parasites that are transferred by this route. Especially tapeworms that require reptiles as definite host use invertebrates as intermediate host. Furthermore, insects, such as flies can function as vectors to allocate parasite eggs to the next host (Schneller et al., 2008). However, the probability of infection might not be equal during the sampling period and might be an additional factor for the later detection of gastrointestinal parasites in *F.* cf. *nicosiai*. Regarding the comparison of the adults of both species per month, we found that *F*. cf. *nicosiai* exhibited a higher prevalence of gastrointestinal parasites apart from May. We suspect that the longer cumulative exposure might have an influence on this observation. The higher prevalence of gastrointestinal parasites in *F. labordi* in May might be attributed to the relatively small amount of fecal samples of *F.* cf. *nicosiai* (n = 10) compared to *F. labordi* (n = 64).

With respect to multiple infections, we observed no significant interspecific differences. Although, triple infections were only found in *F. labordi. Furcifer* cf. *nicosiai*, as the longer living species probably has a comparatively longer exposure to potential infections, might have developed some resistance against these pathogens. However, when entering the mating season, the prevalence of gastrointestinal and blood parasites increased in *F*. cf. *nicosiai* as well. Concerning the intensity of coccidian oocyst shedding, we did not detect interspecific differences. However, as *F*. cf. *nicosiai* is the larger species, similar intensities of coccidian infection probably have milder effects on the individual's body condition.

Within the samples of both species, we found a very low prevalence of oxyurids, which is in accordance to the findings of Lutzmann (2007), who examined fecal samples of several wild living chameleon species from Masoala, Madagascar. Contrary to our findings, these parasites were frequently detected in specimens that were kept in captivity (Biallas, 2013). Probably, in a terrarium, where the home range is very restricted, oxyurid density can increase rapidly due to their direct life cycle and resistant eggs.

In *F*. cf. *nicosiai*, we found a higher prevalence of filariid infection, which could be in turn explained by the comparatively longer exposition to blood-sucking arthropods such as *Culex* and *Aedes* due to their comparatively longer lifespan. Moreover, adult specimen of *F.* cf. *nicosiai* are considerably larger than adults of *F. labordi* and might therefore be easier to detect for mosquitos. As the prepatent period takes approx. 6 months (Széll et al., 2001), due to its shorter lifespan *F. labordi* is less prone to be adversely affected by foleyellosis. Subsequently, this species rather irregularly functions as primary host for *Foleyella* aff. *furcata*. Contrary to *F. labordi*, we found that the comparatively high prevalence in January decreased towards March in *F*. cf. *nicosiai*, but hereafter rises towards June. Initially, this observation might be explained by the small amount of blood samples (n = 3) from *F.* cf. *nicosiai* in January. However, as sample size is respectively higher in the following months, this might indicate some immune defense mechanisms against the parasite, which changes to immunosenescence towards the beginning of the dry season. Additionally, the life cycle of *Foleyella* might also have an influence of the observed pattern. As adult stages are known to predominantly inhabit skin or muscle tissue, an infection with this parasite might not have always been detected.

We found that the prevalence and intensity of mites was higher in *F.* cf. *nicosiai*, which could be caused by their larger average body size and subsequently easier detection for mites. Moreover, regarding the differences in intensity, mite pockets are larger in *F*. cf. *nicosiai* and might therefore offer more space for these ectoparasites.

Concerning interspecific comparison, niche differentiation may in turn result in differences in the exposure to parasites. In our previous study (Eckhardt et al., 2019) we observed that adults of *F*. cf. *nicosiai* showed significant higher roosting sites, which might reflect differences in habitat use of both species. Here, the composition of food insects (vectors for gastrointestinal parasites), mosquitos (vectors for blood parasites) and mites might be unequal.

In total, detailed studies investigating parasite burden and in connection with their life history and seasonality in reptiles are lacking (Zimmerman et al., 2010). However, a comparative study in mammals revealed weak relationships between parasite species richness and longevity (Cooper et al., 2012). These authors found a significant negative relationship between longevity and parasite species richness for ungulates, but not for carnivores or primates, indicating no general pattern of parasite richness according to life history in vertebrates. In contrast to our expectations, we found higher prevalences of gastrointestinal-, blood - and ectoparasites in adult *F*. cf. *nicosiai* compared to adult *F. labordi*. As *F*. cf. *nicosiai* is the longer living and larger species, these observations could be caused by differences of cumulative exposure, as well as body size. Here, it is difficult to disentangle which factors or interplay of factors influence these pattern. However, the fact that juveniles of *F*. cf. *nicosiai* show comparatively low infection rates until their maturation that takes approx. 11 months, suggests some immune defense mechanisms in juveniles compared to *F. labordi*. Although, following maturation this species seems to be affected by serious parasite infections, indicating that this age cohort reallocates their energy investment from self-maintenance to reproduction. Moreover, the accelerated growth rates that we observed after the aestivation (Eckhardt et al., 2019), which involves higher food requirements might additionally influence the raise in gastrointestinal parasites.

### 4.3 Intersexual comparison

In *F. labordi*, males showed a significantly higher prevalence of gastrointestinal parasites and additionally higher intensity in coccidian infestation than females. Similarly, a study of the small marsupial *Antechinus stuartii* found that males, which are the significantly shorter living sex, had remarkably higher prevalence of gastrointestinal parasites at the end of the mating season compared to females (Beveridge and Barker, 1976). Additional studies in lizards (Uller and Olsson, 2003) found that males are more susceptible to parasite infection, which was attributed to the immune-suppressive effects of testosterone, at least during the reproductive period. Among wild vertebrates, the prevalence and intensity of parasitic infections is also generally higher in males than females (Klein, 2000). Here, sex differences in exposure as well as susceptibility to parasites probably contribute to sex-based differences in the intensity and prevalence of parasites. For example, males are more likely to engage in behaviors, such as aggression and dispersal, increasing the likelihood of contact with parasites (Zuk and McKean, 1996; Roberts et al., 2001). Males also often are larger than conspecific females, which may make them more obvious targets for parasites (Zuk and McKean, 1996). Despite differences in the likelihood of exposure, several studies illustrate that harsh intrasexual combats and chronic physiological stress leads to increased susceptibility to infections, which is in accordance with the earlier die-off of in males (Eckhardt et al., 2017). In their review examining the immunocompetence handicap hypothesis, Roberts et al. (2004) suggest that there is at best weak evidence that testosterone directly influences immune function of males.

In contrast to our predictions, we did not find any sex difference in *F. labordi* with respect to filarial infections. As observed in both species, the microfilaria prevalence increased with season, and males of *F*. cf. *nicosiai* showed a higher prevalence than females, perhaps because of their larger body size and/or higher susceptibility.

Moreover, in both species, we detected sex differences in the intensity of acarian infestation, with males housing considerably more mites. This pattern could be caused by the differences in body size and therefore higher amount of blood, which enables larger males to host more mites without higher losses of blood compared to their female conspecifics. Higher intensities of mites in male lizards were also found in a study of Cox and Alder (2007), where males on average carried twice as many mites compared to females. Interestingly, castration reduced mite parasitism to levels comparable to that of females and treatment of castrated males with exogenous testosterone elevated mite counts to levels characteristic of intact males.

### 4.4 *Furcifer labordi* in captivity

When comparing caged individuals with their wild conspecifics, we found that the prevalence of gastrointestinal parasite infection was significantly higher in the latter one. Although we tried to shield captive specimens from infection, they probably took up parasites from the crickets, lepidopterans and orthopterans that were fed to them. These insects might have transferred parasitic eggs or oocysts. Since the caged chameleons were collected from the forest at the age of approximately two months, they might have also taken up parasite stages before. The two males that were infected with coccidians showed obvious senescent declines. Compared to the median survival time of caged males, (8.2 months, Eckhardt et al., 2017), these specimens showed a remarkably shorter lifespan. In contrast to their wild living conspecifics, we did not find significant intersexual differences in survival within the caged animals. Similar results were obtained in mouse lemurs *M. murinus*, where survival in the wild was strongly female-biased (Kraus et al., 2008; Languille et al., 2012), whereas longevity in captivity was slightly male-biased (Perret, 1997). These findings support the previously mentioned suggestion of Roberts et al. (2004) that testosterone alone is unlikely to be responsible for accelerated senescence and die off in males in the wild. In total, as the caged chameleons were shielded from predation, starvation, desiccation and at least partly from infections, it is not possible to pinpoint the factors facilitating their longer survival in captivity. To identify to which extant the presence or absence of parasites influence the lifespan of caged animals, an experimental manipulation of parasite burden could give insight into the direct effect of parasite infection.

## 5 Conclusions

Our study provides rare information about the age-related patterns of health in the shortest living tetrapod species in the wild and suggest that *F. labordi* rather relies on nonspecific and inflammatory immune defenses than on acquired immunity. Moreover, as vertebrates obligatorily develop an adaptive immune system, we assume a downregulation of the acquired immunity with age. As the prevalence in parasites increased comparatively later in *F*. cf. *nicosiai*, we suspect that this species invests more energy in the development of an adaptive immune system until maturation. However, adults of both species seem to suffer from parasite infections. The parasite burden observed in fecal and blood samples revealed the combined outcome of several factors: the parasites encountered by the host, when the parasite matured and reproduced within the host, and how effective the host's immune system was in preventing or eliminating the infection. While it is difficult to disentangle these factors, we found males having higher parasite infection rates than females, and the older animals to suffer from the higher parasite burden, suggesting a downregulation of the acquired immunity in both species. Finally, the conduction of cage experiments including both species and sexes with a controlled manipulation of parasite burden could clarify the direct influence of the effect of parasites on the lifespan of both species.

## Declaration of competing interest

All authors from the manuscript disclose any financial and personal relationships with other people or organizations that could inappropriately influence our work.
